# A Case-Control Study of the Dose-Response Relationship Between Thrombin Activatable Fibrinolysis Inhibitor and Acute Myocardial Infarction

**DOI:** 10.3389/fcvm.2022.823381

**Published:** 2022-02-28

**Authors:** Mengnan Zhao, Dan Zhao, Yuning Li, Xiaonan Wang, Boyu Yi, Bo Zhou

**Affiliations:** ^1^Department of Clinical Epidemiology and Evidence-Based Medicine, The First Affiliated Hospital, China Medical University, Shenyang, China; ^2^Liaoning Provincial Center for Disease Control and Prevention, Shenyang, China

**Keywords:** TAFI, case-control study, dose-response relationship, AMI, risk factor

## Abstract

**Background:**

Acute myocardial infarction (AMI) is considered an acute coronary syndrome (ACS), which is caused by the death of myocardial cells after prolonged ischemia, and there is a high risk of sudden death during AMI. Therefore, the purpose of this study is to explore the relationship between thrombin activatable fibrinolysis inhibitor (TAFI) and AMI and provide evidence for their association and potentially the prevention of AMI.

**Methods:**

There were 228 subjects included in this retrospective study, which included 78 AMI patients and 150 controls. The immune turbidimetry was used to measure TAFI concentration in the serum. Mann–Whitney U test was used to compare serum TAFI levels. The logistic regression analysis was used to construct a model of influencing factors of AMI. The dose-response relationship between serum TAFI level and AMI was explored by using the restricted cubic spline (RCS) functions combined with logistic regression analysis.

**Results:**

The serum TAFI levels of the AMI group were higher than the control group’s (*P* = 0.003). The risk of AMI in the high-TAFI level group was 2.24 times higher than the low-TAFI level group (*P* = 0.007) and it was 2.74 times higher after adjustment of other risk factors (*P* = 0.025). According to the dose-response curve, the risk of AMI increased significantly with an increase of serum TAFI concentration (*P* = 0.0387).

**Conclusion:**

Acute myocardial infarction patients had higher serum TAFI levels, and TAFI was an independent risk factor for AMI patients. Serum TAFI levels demonstrated a dose- dependent response to the risk of AMI. Our study provides evidence that TAFI could be used for risk stratification of AMI patients.

## Background

Coronary heart disease is a major cause of death worldwide and the burden of AMI remains high despite optimal therapies (1). Cardiovascular death is the leading cause of death in the United States, with a mortality rate more than double of all cancer mortality. More than 50% of these cardiovascular deaths are due to acute myocardial infarction (2). In China, the mortality rate of AMI is continued on the rise from 2002 to 2016. Since 2005, the mortality rate of AMI has shown a sharp upward trend, especially in rural areas. In 2016, the mortality rate of AMI was 58.69 per 100,000 in metropolitan areas and 74.72 per 100,000 in rural areas (3). The increasing social and economic burden of AMI has become an important public health problem, which needs to be addressed and prevented urgently.

The AMI occurs when myocardial perfusion is reduced, which is most commonly caused by atherosclerotic plaque rupture and coronary thrombosis (4). TAFI is a fibrinolysis inhibitor in the form of plasminogen and plays an important role in the regulation of the coagulation cascade and the fibrinolysis system (5). Dysregulation of TAFI has been associated with many thrombotic diseases (6). The activated TAFI removes the lysine residues from the carboxyl end of the partially degraded fibrin (7), thus preventing the feedback loop of fibrinolytic cascade trigger by tissue plasminogen activator (8, 9). Therefore, the physiological function of TAFI is anti-fibrinolysis. In addition, many studies have shown that circulating TAFI level is associated with coronary artery disease progression and could be used as a biomarker in clinical practice (10). However, the relationship between TAFI levels and AMI is controversial and relatively few studies have been conducted. We hypothesize that TAFI is an independent risk factor for AMI patients and that Serum TAFI concentration has a dose-dependent response to the risk of AMI. Therefore, this study aims to use the case-control study method and analyze the relationship between TAFI levels in serum and AMI with the expectation that such study can provide the basis for making the prevention and prophylactic measures of AMI.

## Materials and Methods

### Participants

All subjects were from the same hospital in Liaoning Province, China. In total, 78 AMI patients and 222 controls were collected in this retrospective study. Inclusion criteria were as follows: the hospital confirmed the diagnosis of acute myocardial infarction patients and the time of diagnosis was between April 2018 to September 2019. Exclusion criteria were as follows: patients with other comorbidities such as liver and kidney disease, coagulation disorder, pulmonary embolism, cancer, and patients with confirmed pregnancy. Control cases were referred to the non-acute myocardial infarction patients in the same hospital at the same duration. Ethics approval was obtained from the China Medical University Ethics Committee [(2019) Approval No. 287].

### Case-Control Matching Method and Sample Size Calculation

In order to balance the distribution of age and sex in cases and controls, propensity score matching (PSM) was used to match the included individuals (11). The nearest neighbor matching was used and the width of the calipers in matching was 0.02. The ratio between the case group and control group was 1:2. The package “MatchIt” in R 4.1.1 was used to implement the process of PSM (12). Then the sample size for case-control study was calculated using PASS 11 software (13). In sample size calculation, α was set as 0.1 and β was set as 0.15 (“α” is the probability of rejecting a true null hypothesis that was desired, “β” is the probability of accepting a false null hypothesis).

### Blood Sample Collection

Serum samples from participants meeting the inclusion criteria in the same hospital were collected during April 2018 to September 2019. The samples were the surplus serum stored by the laboratory department for in-patient testing. Blood samples were collected after more than 8 h fast and 1 mL serum was extracted into the EP tube. The blood samples were placed in a refrigerator at −80°C, and then tested together after all the samples were collected.

### Measurement of Biomarker

Biochemical markers included total cholesterol (TC), triglyceride (TG), high-density lipoprotein (HDL), low-density lipoprotein (LDL), Sodium (Na^+^), potassium (K^+^), glucose (Glu), uric acid (UA), and creatine kinase (CK), all of them were determined by the AU5800 biochemical analyzer (Beckman Coulter, Brea, CA, United States).

The serum TAFI levels were measured by immune turbidimetry using kits provided by Liaoning Maidi Biological Technology and analyzed by the AU480 automatic biochemical analyzer (Beckman Coulter, Brea, CA, United States).

### Statistical Methods

All data were analyzed using SPSS 23.0 and SAS 9.4. The normal distribution of variables was checked by the Kolmogorov–Smirnov test and Q-Q plots. We used the median, upper, lower quartiles, and percentage to describe variables. The Mann–Whitney U test was used to compare the serum TAFI levels between the case group and the control group. The logistic regression analysis was used to construct a model of influencing factors of AMI. The variables were screened by univariate logistic regression, and then variables with *p*-value < 0.10 were included in the multivariate regression analysis. Restricted cubic splines (RCS) with knots at the 5th, 25th, 50th, 75th, and 95th percentiles of the distribution were used to explore the dose-response relationship between serum TAFI concentration and AMI, with the 75th percentile of the control group as the reference point (14). The significance level was *p*-value < 0.05 (2-sided).

## Results

**FIGURE 1:**
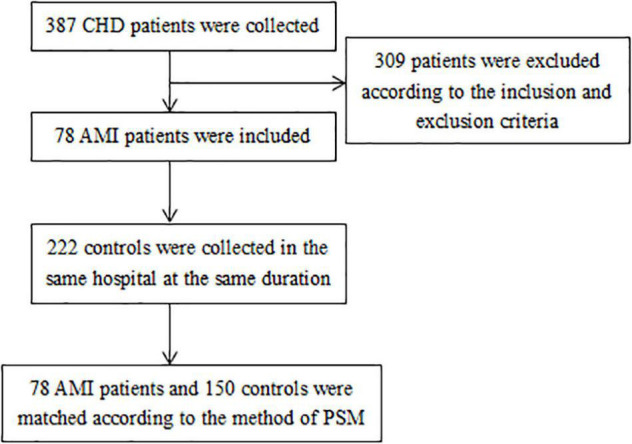
Flow chart of selecting participants.

A total of 78 AMI cases were selected in this study, and 150 controls were matched to the cases in sex and age by the method of PSM. The details of selecting participants were shown in Figure 1. According to the pre-experimental data, serum TAFI concentration exceeding the upper quartile (75th percentiles) of control group was defined as high TAFI level. So serum TAFI concentration higher than 22.76 μg/mL was the main exposure factor observed. The exposure rate in the control population was 25%. The calculation result of OR was 2.23. The estimated sample sizes for case and control group were 76 and 152, respectively. The average age of the AMI group was 61 years, range from 35 to 88 years. The average age of the healthy control group was 58 years, range from 33 to 87 years. Among the 171 males in the study, there were 59 males in the AMI group and 112 males in the control group.

In our results, the median TAFI level of the AMI group was 21.99 μg/ml, the upper and lower quartile was 26.13 and 18.86 μg/ml, respectively. The median, upper and lower quartiles of TAFI levels in the control group were 20.78, 22.76, and 17.35 μg/ml, respectively. Overall, the serum TAFI level in the AMI group was higher than in the control group (*P* = 0.003, Mann–Whitney U test).

The upper quartile of serum TAFI concentration in the control group was used as the baseline (=22.76 μg/ml), and all the subjects were divided into low TAFI level group (≤22.76 μg/ml) and high TAFI level group (>22.76 μg/ml). At the same time, the low TAFI level group was used as a reference point for further analysis.

After univariate logistic regression analysis for AMI, our results showed that smoke, alcohol, hyperglycemia, high CK, and high TAFI are the risk factors of AMI (*P* < 0.05). On the contrary, high K^+^ may be a protective factor of AMI (*P* = 0.01) (Table 1).

**TABLE 1:** Univariate logistic regression for acute myocardial infarction (AMI).

Variate	Cases [*n* (%)]	Controls [*n* (%)]	*OR*	95%*CI*	*P*
Ethnic Han	69 (88.46)	131 (87.33)	1.11	0.48–2.59	0.806
Retiree	42 (53.85)	73 (48.67)	1.23	0.71–2.13	0.458
Smoke	44 (56.41)	38 (25.33)	3.81	2.14–6.81	<0.001
Alcohol	40 (51.28)	38 (25.33)	3.10	1.74–5.52	<0.001
Overweight	51 (65.38)	80 (53.33)	1.65	0.94–2.91	0.082
High TC	13 (16.67)	22 (14.66)	1.16	0.55–2.46	0.691
High TG	23 (29.49)	30 (20.00)	1.67	0.89–3.14	0.109
High HDL	4 (5.13)	14 (9.33)	0.53	0.17–1.65	0.271
High LDL	10 (12.82)	30 (20.00)	0.59	0.27–1.28	0.180
Hyperglycemia	37 (47.44)	41 (27.33)	2.40	1.35–4.25	0.003
High Na^+^	45 (57.69)	98 (65.33)	0.72	0.41–1.27	0.258
High K^+^	26 (33.33)	77 (51.33)	0.47	0.27–0.84	0.010
High UA	13 (16.67)	21 (14.00)	1.25	0.59–2.66	0.559
High CK	67 (85.90)	29 (19.33)	27.83	12.84–60.34	<0.001
High TAFI	33 (42.31)	37 (24.67)	2.24	1.25–4.01	0.007

*Overweight: BMI ≥ 24 kg/m^2^; High TC: TC ≥ 5.72 mmol/L; High TG: TG ≥ 1.89 mmol/L; High HDL: HDL ≥ 1.55 mmol/L; High LDL: LDL ≥ 3.60 mmol/L; Hyperglycemia: blood glucose ≥ 6.11 mmol/L; High Na^+^: Na^+^ ≥ 139.00 mmol/L; High K^+^: K^+^ ≥ 4.03 mmol/L; High UA: UA ≥ 440.00 μmol/L; High CK: CK ≥ 196.00 U/L; High TAFI: TAFI > 22.76 μg/mL.*

Further to this, considering the results of univariate logistic regression, we adjusted smoke, alcohol consumption, BMI, Glu, K^+^, CK, and TAFI as possible influencing factors (*P* < 0.10). For multivariate regression analysis, our data showed that smoke, high CK, and high TAFI are the independent risk factors of AMI (*P* < 0.05) (Table 2). Multivariate logistic regression showed that high TAFI level (>22.76 μg/ml) was significantly associated with the risk of AMI (OR: 2.74, 95% CI: 1.14–6.60).

**TABLE 2:** Multivariate logistic regression for AMI.

Variate	β	*s_‘*x*_*	Wald χ^2^	OR	95%CI	*P*
Smoke	1.468	0.427	11.849	4.34	1.88–10.0	<0.001
High CK	3.573	0.450	63.125	35.61	14.75–85.95	<0.001
High TAFI	1.008	0.449	5.044	2.74	1.14–6.60	0.025

*High CK: CK ≥ 196.00 U/L; High TAFI: TAFI > 22.76 μg/mL.*

**FIGURE 2:**
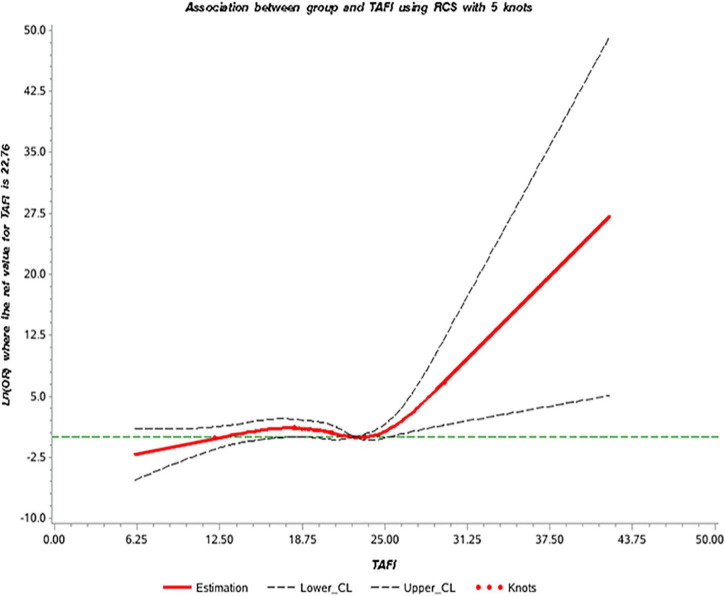
The dose-response curve of the relationship between thrombin activatable fibrinolysis inhibitor (TAFI) and acute myocardial infarction (AMI).

We selected five data points (p5, p25, p50, p75, and p95) of TAFI levels to construct the dose-response relationship model and used the 75th percentile of the control group (p75, 22.76 μg/ml) as the reference point (10). Our data showed that the association between TAFI levels and AMI was non-linear (*P* = 0.0266) and the correlation between TAFI levels and AMI was statistically significant (*P* = 0.0387). The dose-response curve demonstrated that the risk of AMI (odds ratio) increased significantly with the increase of serum TAFI concentration (Figure 2).

## Discussion

In recent years, it has been found that TAFI is associated with inflammation and progression of arteriosclerotic coronary artery disease (5). TAFI levels are genetically controlled and its increase is associated with thrombogenesis (15), which has a unique role in maintaining hemostasis, and TAFI could be a risk factor for thrombotic disease (16). There is a persistent hypercoagulable status in AMI patients, and TAFI concentration is assumed to be related to the risk of AMI. The anti-fibrinolysis function of TAFI depends mainly on the TAFI concentration and the rate of its activation (17).

Our study used the state of the art immune turbidimetry to measure the serum TAFI concentration, which provides high accuracy than the traditional method. The median TAFI level of the AMI group was 21.99 μg/ml and was higher than in the control group. In an observational prospective study, higher concentrations of TAFI were also associated with poorer cardiac reperfusion and disease recurrence outcomes in AMI patients treated with fibrinolysis (18). It has been reported that TAFI contributed to thrombosis and inflammation cascade, therefore, TAFI can be used as a biomarker for the diagnosis or risk prediction of the acute coronary syndrome (ACS). However, other studies have shown that the serum TAFI levels in ACS patients were significantly lower than in the control group (19, 20) and a higher level of TAFI may protect against MI (21). This discrepancy may be due to the differences in TAFI measurement methods, patients’ selection criteria, and blood collection time points. In addition to the immunoturbidimetry used in this study, high-performance liquid chromatography (HPLC)-assisted assay was also used to measure serum TAFI concentration in other studies (20, 22). Different measurement methods have their inherent advantages and disadvantages. In terms of cases’ selection, most of the studies included AMI cases consisting of both STEMI (ST segment elevation myocardial infarction) and NSTEMI (non-ST segment elevation myocardial infarction) patients, which reduced the error of the studies (19, 20). The difference in age and sex between the case and control groups may affect results of these studies. Therefore, the distribution of age and sex in the two groups should be similar.

Our data using univariate and multivariate logistic regression analysis and showed that higher TAFI levels increased the risk of AMI (Univariate: OR = 2.24, 95% CI = 1.25, 4.01; Multivariate: OR = 2.74, 95% CI = 1.14, 6.60), which suggested that TAFI was a risk factor for AMI. The proposed mechanism of TAFI is likely involved in the process of endothelium damage, platelets adhesion, and aggregation, forming thrombosis and atheromatous plaque progression (23). The dose-response curve showed that the risk of AMI increased significantly with an increase of serum TAFI concentration. It may be due to the coagulation cascade and antifibrinolytic effects of TAFI. When blood vessels are damaged, vessels and endothelium are stimulated to release thrombin, which in turn activate TAFI to inhibit fibrinolysis and lead to thrombosis. Although our data demonstrated the association of TAFI and AMI, it should be noted here that our total sample number is small and this study is a single-center retrospective study therefore the data may be under representative of the overall population. Currently, the diagnosis of AMI is mainly based on electrocardiogram, myocardial zymogram and the level of troponin, the aim of this study was to improve the diagnostic accuracy of AMI by adding the TAFI to the list.

This study used a variety of statistical methods to analyze the association between TAFI level and AMI, we concluded that the high TAFI concentration is a risk factor for AMI. At the same time, the concentration of TAFI in serum was measured by immune turbidimetry, which is a simpler method and effectively reduce the measurement error due to strict stand operation procedure. However, the case group and control group in this study were only from one hospital, and could not represent the whole target population, so they were prone to hospitalization rate bias and the time sequence of causality between AMI and serum TAFI level could not be definiteness, therefore, our study warrant a further study that explore the causal relationship between TAFI and AMI in a multi-center prospective trial.

## Conclusion

In conclusion, our study found that high serum TAFI levels could be an independent risk factor for AMI, therefore, serum TAFI level could be a biomarker for the diagnosis of AMI. Our observational study demonstrates the dose-dependent relationship between serum TAFI levels and AMI and provides evidence for using TAFI as a biomarker for risk stratification of AMI patients.

## Data Availability Statement

The raw data supporting the conclusions of this article will be made available by the authors, without undue reservation.

## Ethics Statement

The studies involving human participants were reviewed and approved by the China Medical University Ethics Committee [(2019) Approval No. 287], China Medical University. Written informed consent for participation was not required for this study in accordance with the national legislation and the institutional requirements.

## Author Contributions

MZ proposed the idea for the study and wrote the manuscript. DZ revised the manuscript and designed the tables. YL, XW, and BY contributed to the design of study and analyzed the data. BZ revised the manuscript and supervised the whole study. All authors contributed to manuscript revision, read, and approved the submitted version.

## Conflict of Interest

The authors declare that the research was conducted in the absence of any commercial or financial relationships that could be construed as a potential conflict of interest.

## Publisher’s Note

All claims expressed in this article are solely those of the authors and do not necessarily represent those of their affiliated organizations, or those of the publisher, the editors and the reviewers. Any product that may be evaluated in this article, or claim that may be made by its manufacturer, is not guaranteed or endorsed by the publisher.
